# Correction: Burden of five healthcare associated infections in Australia

**DOI:** 10.1186/s13756-022-01167-y

**Published:** 2022-11-01

**Authors:** M. J. Lydeamore, Mitchell B. G, T. Bucknall, A. C. Cheng, P. L. Russo, A. J. Stewardson

**Affiliations:** 1 Department of Infectious Diseases, The Alfred and Central Clinical School, Monash University, Melbourne, VIC Australia; 2 School of Nursing and Midwifery, University of Newcastle, Ourimbah, NSW Australia; 3 School of Nursing, Avondale University, Cooranbong, NSW Australia; 4 School of Nursing and Midwifery, Deakin University, Geelong, VIC Australia; 5 Deakin Centre for Quality and Patient Safety Research-Alfred Health Partnership, Melbourne, VIC Australia; 6 School of Nursing and Midwifery, Monash University, Frankston, VIC Australia; 7 Department of Nursing Research, Cabrini Institute, Malvern, VIC Australia

Upon publication [1], the authors noted an error in Figs. 1, 2, 3 and 4. The corrected figures are below. This error related only to stratification of results by age group category resulting in a change to the x-axis scale, and does not otherwise impact on the results of this paper.

**Figure 1:**
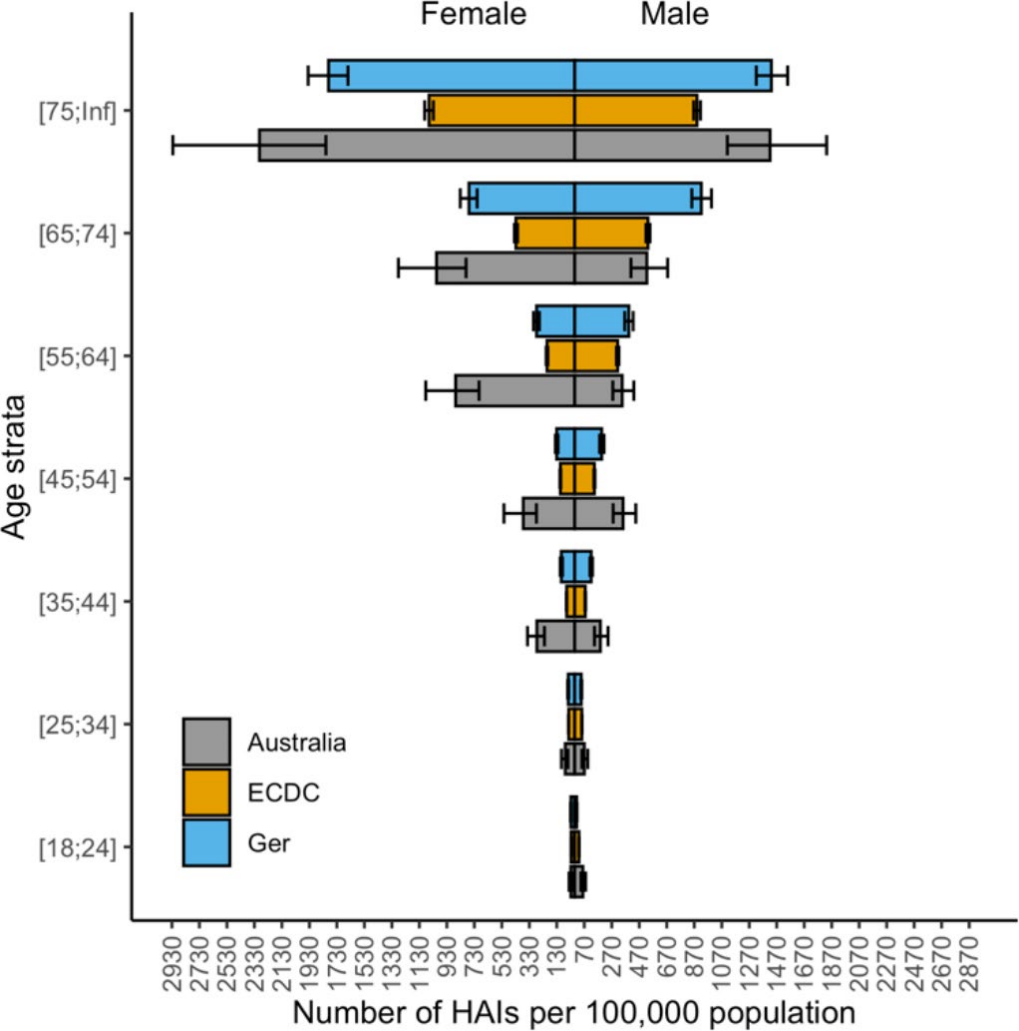
Figure 1 Number of cases of healthcare associated infections per 100,000 population in Australia, presented with previously published data from the EU and Germany

**Figure 2:**
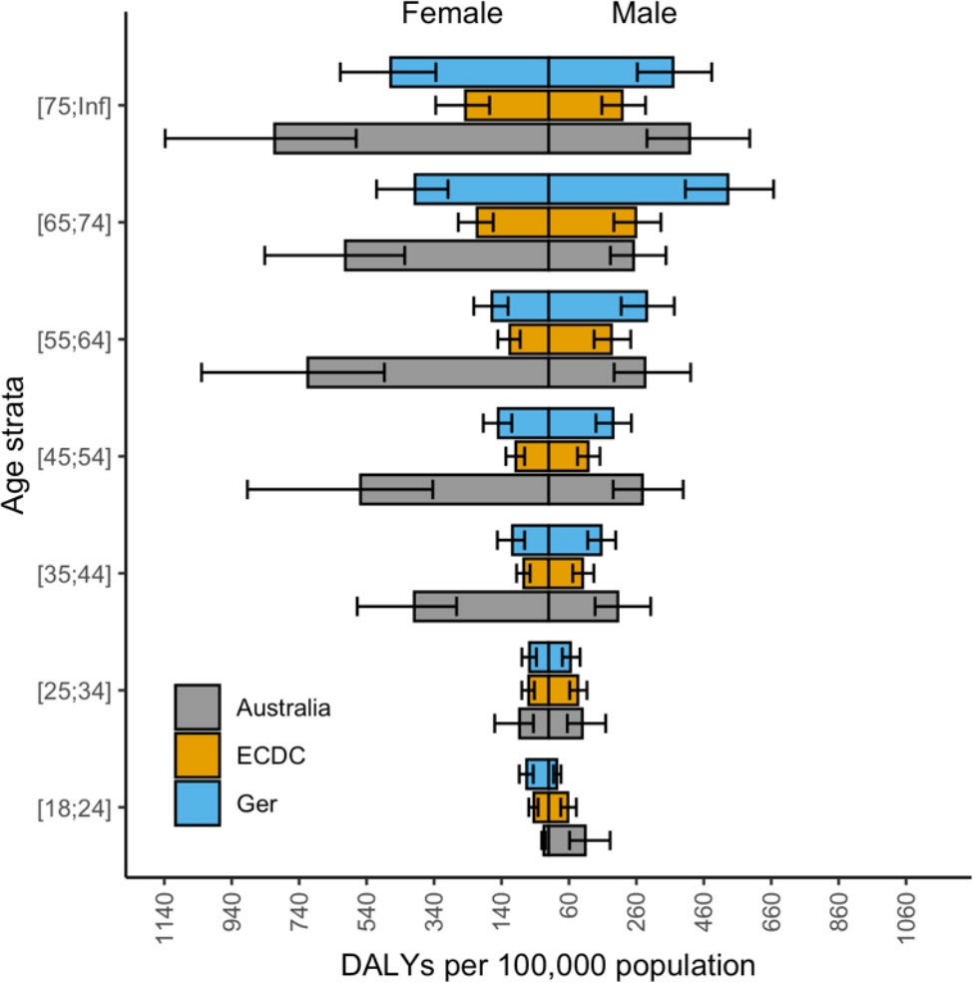
Figure 2 Number of DALYs from healthcare associated infections, stratified by age, in Australia, presented with previously published data from the EU and Germany, normalised by population

**Figure 3:**
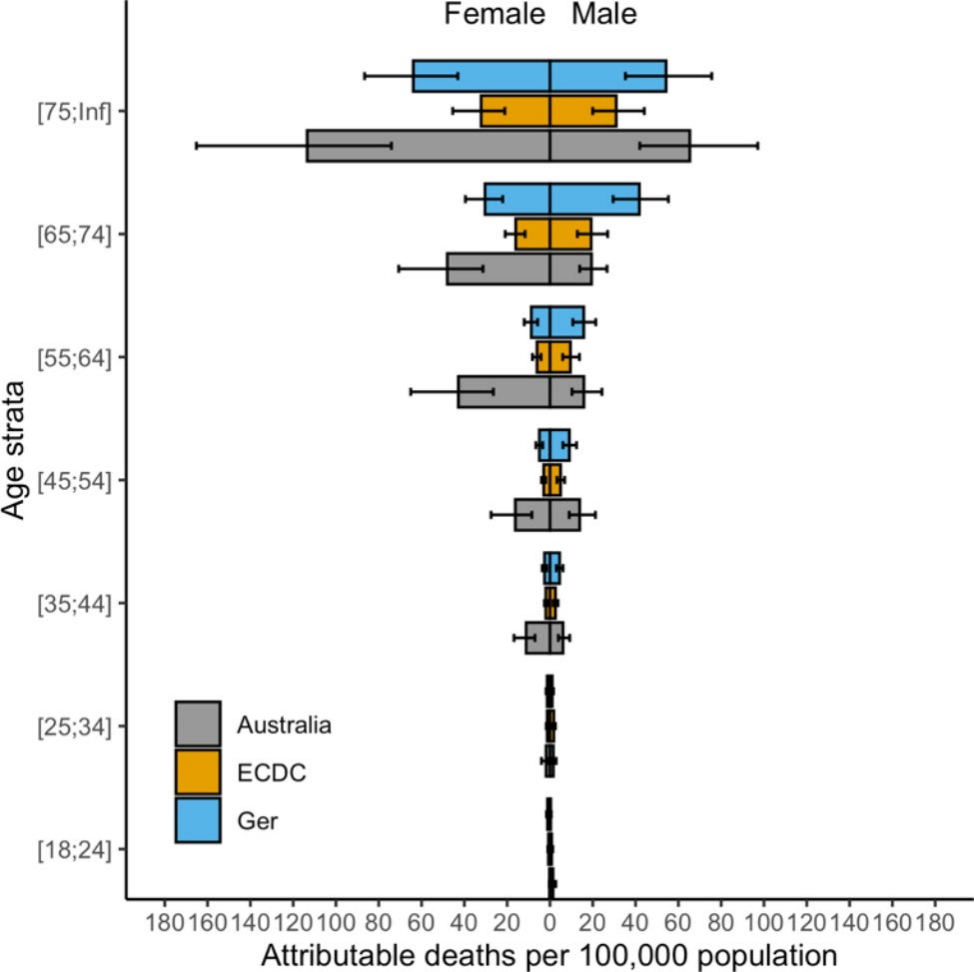
Figure 3 Number of attributable deaths from healthcare associated infections, stratified by age, in Australia, presented with previously

**Figure 4:**
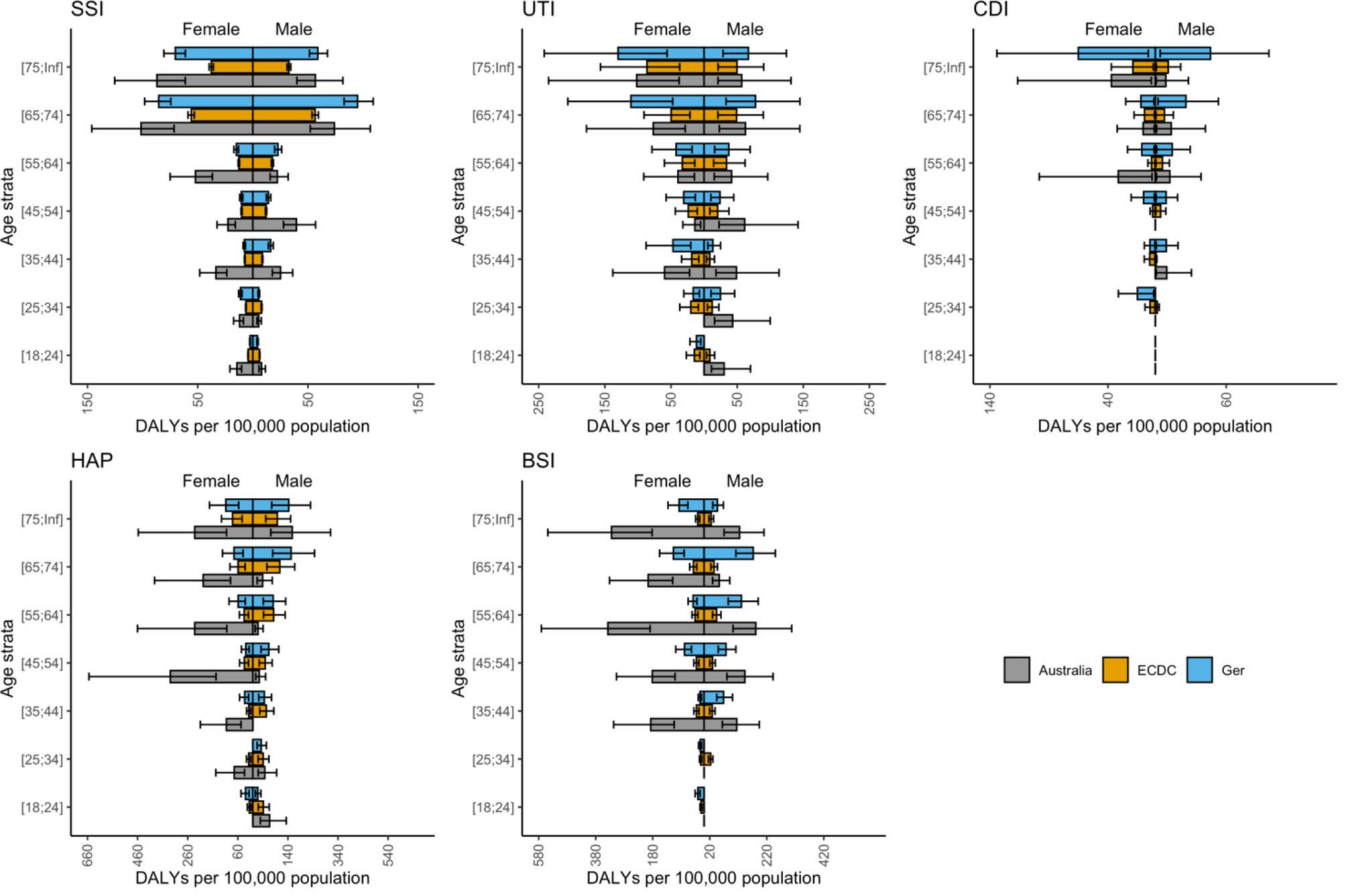
Figure 4 Estimated rate of five healthcare associated infections, normalised by population, in Australia, presented with previously published data from Europe and Germany
